# Human Cytomegalovirus Immediate Early 86-kDa Protein Blocks Transcription and Induces Degradation of the Immature Interleukin-1β Protein during Virion-Mediated Activation of the AIM2 Inflammasome

**DOI:** 10.1128/mBio.02510-18

**Published:** 2019-02-12

**Authors:** Sara Botto, Jinu Abraham, Nobuyo Mizuno, Kara Pryke, Bryan Gall, Igor Landais, Daniel N. Streblow, Klaus J. Fruh, Victor R. DeFilippis

**Affiliations:** a Vaccine and Gene Therapy Institute, Oregon Health and Science University, Portland, Oregon, USA; Stony Brook University

**Keywords:** AIM2, DNA sensor, HCMV, IE2, IE86, STING, cGAS, cytomegalovirus, inflammasome, innate immunity, interferons

## Abstract

Secretion of interleukin-1β (IL-1β) represents a fundamental innate immune response to microbial infection that, at the molecular level, occurs following activation of proteolytic caspases that cleave the immature protein into a secretable form. Human cytomegalovirus (HCMV) is the archetypal betaherpesvirus that is invariably capable of lifelong infection through the activity of numerous virally encoded immune evasion phenotypes. Innate immune pathways responsive to cytoplasmic double-stranded DNA (dsDNA) are known to be activated in response to contact between HCMV and host cells. Here, we used clustered regularly interspaced short palindromic repeat (CRISPR)–CRISPR-associated protein 9 (Cas9) genome editing to demonstrate that the dsDNA receptor absent in melanoma 2 (AIM2) is required for secretion of IL-1β following HCMV infection. Furthermore, dsDNA-responsive innate signaling induced by HCMV infection that leads to activation of the type I interferon response is also shown, unexpectedly, to play a contributory role in IL-1β secretion. Importantly, we also show that rendering virus particles inactive by UV exposure leads to substantially increased IL-1β processing and secretion and that live HCMV can inhibit this, suggesting the virus encodes factors that confer an inhibitory effect on this response. Further examination revealed that ectopic expression of the immediate early (IE) 86-kDa protein (IE86) is actually associated with a block in transcription of the pro-IL-1β gene and, independently, diminishment of the immature protein. Overall, these results reveal two new and distinct phenotypes conferred by the HCMV IE86 protein, as well as an unusual circumstance in which a single herpesviral protein exhibits inhibitory effects on multiple molecular processes within the same innate immune response.

## Abstract (precis)

Persistent infection with HCMV is associated with the operation of diverse evasion phenotypes directed at antiviral immunity. Obstruction of intrinsic and innate immune responses is typically conferred by viral proteins either associated with the viral particle or expressed immediately after entry. In line with this, numerous phenotypes are attributed to the HCMV IE86 protein that involve interference with innate immune processes via transcriptional and protein-directed mechanisms. We describe novel IE86-mediated phenotypes aimed at virus-induced secretion of IL-1β. Intriguingly, while many viruses target the function of the molecular scaffold required for IL-1β maturation to prevent this response, we find that HCMV and IE86 target the IL-1β protein specifically. Moreover, we show that IE86 impairs both the synthesis of the IL-1β transcript and the stability of the immature protein. This indicates an unusual phenomenon in which a single viral protein exhibits two molecularly separate evasion phenotypes directed at a single innate cytokine.

## INTRODUCTION

The innate immune response represents one of the first and most rapid host defense barriers against infecting microbes. Pattern recognition receptors (PRRs) are initially responsible for detecting pathogen- and danger-associated molecular patterns (PAMPs and DAMPs, respectively) and thereby activating innate signaling processes that culminate in the expression of antiviral effectors and secretion of immunologically active factors (reviewed in references 1 and 2). Among these are interleukin-1β (IL-1β) and IL-18, which are synthesized as immature proteins (pro-IL) that are then cleaved by the cysteine protease caspase-1. Maturation of caspase-1 itself requires the assembly of an intracellular multiprotein complex termed the inflammasome that consists of an initiating PRR, the procaspase-1 zymogen, and the adaptor molecule apoptosis-associated speck-like molecule containing a caspase recruitment domain (ASC) (3, 4). PRRs capable of initiating DAMP- and PAMP-dependent inflammasome activation include multiple distinct NOD-like receptors (NLRs) (5), as well as the cytoplasmic receptors of double-stranded DNA (dsDNA) interferon gamma (IFN-γ)-inducible protein 16 (IFI16) and absent in melanoma 2 (AIM2) (6–11). Importantly, two distinct innate signals are generally required for full induction of the inflammasome. A priming step involves activation of transcriptional processes via nuclear factor κB (NF-κB) that result in the synthesis of mRNAs encoding caspase-1 and pro-IL-1β, as well as other components of the inflammasome, such as PRRs (12–14). The second signal comprises PRR-induced assembly of the multiprotein complex and maturation of caspase-1, which leads to processing of pro-IL-1β, pro-IL-18, and gasdermin D (GSDMD), a pore-forming protein required for extracellular release of mature IL-1β and IL-18 (15–17). Ultimately, inflammasome-processed cytokines contribute to the antiviral response by establishing proinflammatory tissue states that promote the recruitment and activation of immune cells (18, 19) and antiviral cellular states (20), as well as by causing pyroptotic cell death (reviewed in reference 21).

Human cytomegalovirus (HCMV) is a ubiquitous and species-specific betaherpesvirus that invariably infects with lifelong persistence. This is accomplished largely through multifaceted and sophisticated phenotypic mechanisms of immune evasion (reviewed in reference 22). While the virus is largely asymptomatic in healthy individuals, it can cause severe disease in immunocompromised and immunodeficient populations (22). Furthermore, HCMV is a common cause of birth defects in women experiencing primary infection or reactivation during organogenesis (23). Persistent HCMV infection is also strongly associated with the development of cardiovascular diseases like atherosclerosis (24). Importantly, any potential links between HCMV infection and virus-induced mechanisms of chronic inflammation that likely contribute to pathogenic outcomes like cardiovascular dysfunction remain poorly examined research foci. Ultimately, however, HCMV-associated disease develops due to the ability of the virus to infect and persist in the host and its ability to impair and circumvent the host immune system (25).

Exposure of cells to HCMV *in vitro* strongly activates innate immune signaling, as indicated by the expression of type I and II interferons (26, 27) and proinflammatory cytokines (26, 28–30) and the secretion of IL-1β (31–33), although these responses often vary based on cell type (34). The mechanistic bases of HCMV-mediated stimulation of signaling pathways that lead to activation of transcription factors IFN regulatory factor 3 (IRF3) and NF-κB, which are involved in the synthesis of antiviral and proinflammatory mRNAs, respectively, have been studied in detail, and crucial roles for dsDNA-dependent PRRs, such as ZBP1/DAI, cGAS, and IFI16, have been reported (35–40). However, many unanswered questions remain regarding the induction and function of inflammasome-mediated responses to HCMV infection. While evidence suggests that AIM2 is involved in HCMV-induced processing of IL-1β (41) and that caspase-1 contributes to secretion of the protein from endothelial and smooth muscle cells (33), whether other PRRs or noncanonical inflammasome components are required in myeloid-derived cells, the primary target of latent HCMV infection, has not been reported. Furthermore, the type I IFN and inflammasome systems exist in a complicated and not fully elucidated coregulatory relationship, especially with regard to dsDNA-induced responses (40–42). Potent IFN induction occurs in response to HCMV by way of dsDNA-dependent signaling, and whether these processes confer any directional effects on inflammasome activity remains an important yet unexamined question. Additionally, our understanding of how IL-1β impacts HCMV replication is also lacking. Acute *in vitro* HCMV growth is inhibited by IL-1β (42), possibly by a mechanism that enhances IFN secretion (43). However, HCMV reactivation is promoted in the context of inflammation, such as after tissue transplant (44) or during sepsis (45). Furthermore, *in vivo* murine CMV models showed virus reactivation following direct administration of IL-1β (46). Interestingly, HCMV has evolved mechanisms to impair IL-1β-dependent signaling (47, 48), as well as NF-κB-dependent transcription (49), suggesting the existence of selective pressure, perhaps derived from antiviral effects of these innate processes. Surprisingly, whether the virus exhibits phenotypes that directly impair the synthesis, processing, or release of IL-1β or the caspase-1 inflammasome has been underexplored.

In this study, we utilized a combination of genome editing by clustered regularly interspaced short palindromic repeat (CRISPR)–CRISPR-associated protein 9 (Cas9) technology and inducible transgene expression to explore functional roles for host and viral proteins in the activation and inhibition of HCMV-induced inflammasome-mediated outcomes. We describe observations that shed novel light on the interaction between HCMV-mediated innate immune activation, inflammasome function, and viral impairment of these processes. Importantly, our work reveals an undescribed viral inhibitory phenotype affecting IL-1β and indicates an additional role for the HCMV intermediate early 86-kDa protein (IE86).

## RESULTS

### Myeloid-derived cells process and secrete IL-1β in response to live or inactivated HCMV.

**FIG 1:**
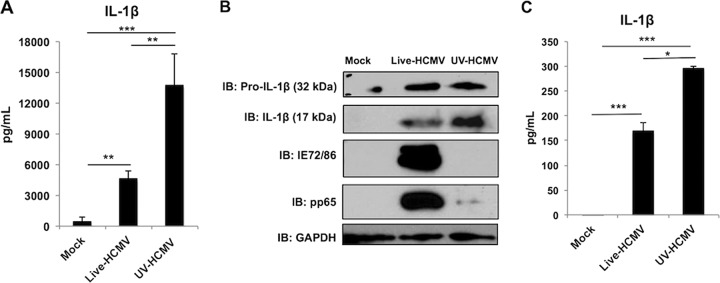
Exposure of differentiated THP-1 cells to live and UV-inactivated HCMV induces secretion of IL-1β. THP-1 cells were differentiated using PMA for 24 h and either left untreated (mock) or exposed to live or UV-inactivated HCMV-TB40/E (MOI of 3) for 18 h. (A) IL-1β secretion in culture medium was measured by ELISA. Values are averages ± standard errors of the means (SEM) from three biological replicates. **, *P* < 0.01; ***, *P* < 0.001. (B) Immunoblot (IB) assay showing results for pro-IL-1β, mature processed IL-1β (supernatant), HCMV IE72/IE86, pp65, and GAPDH. (C) Primary monocytes were differentiated using 2.5 mg/ml M-CSF for 6 days and then stimulated with IFN-γ and LPS for 24 h and either left untreated (mock) or exposed to live or UV-inactivated HCMV-TB40/E (MOI of 10) for 18 h. IL-1β secretion in culture medium was measured by ELISA. Values are averages ± SEM from three biological replicates. One-way analysis of variance (ANOVA) with Bonferroni correction was performed. *, *P* < 0.05; ***, *P* < 0.001.

Given the established importance of dsDNA-reactive PRR pathways to terminate in IFN activation (IFN-terminal) in response to HCMV infection (37–40) and the ability of cytoplasmic dsDNA, as well as other herpesviral PAMPs, to activate the inflammasome (9, 10, 50, 51), we decided to explore this using the promonocytic human cell line THP-1. These cells were first differentiated into a macrophage-like phenotype using phorbol 12-myristate 13-acetate (PMA) as a priming step (52). Differentiated THP-1 cells were then mock treated or exposed to either live HCMV or particles irradiated with UV (UV-HCMV), which abrogates viral transcriptional activity yet allows intracellular release of virion-associated factors (26). We first asked whether UV treatment of these cells led to secretion of IL-1β. As shown by the results in Fig. 1A, relative to mock treatment, significant quantities of IL-1β had been released at 18 h postinfection in response to infection with live HCMV. Intriguingly, virus exposed to UV elicited secretion of the cytokine at levels that were significantly higher than the secretion levels with either mock treatment or live virus exposure. In both cases, secretion of IL-1β was lower at 6 h and 36 h posttreatment (not shown), indicating that the 18-h time point represents a peak response. Since conventional secretion of IL-1β first involves proteolytic processing of the 32-kDa immature pro-IL-1β protein into a mature 17-kDa form, we next examined whether this is also observable following treatment with live or UV-HCMV. As shown by the results in Fig. 1B, the mature 17-kDa form of IL-1β is seen in these cultures following treatment with live HCMV, in contrast to those that were mock treated. Interestingly, exposure to UV-HCMV appeared to trigger a greater accumulation of processed IL-1β (Fig. 1B), a finding consistent with increased secretion of the mature protein (Fig. 1A).

We next examined whether this response was observed in primary human cells of similar derivation. For this, we isolated primary monocytes from whole blood by double gradient centrifugation. These were differentiated into M0 macrophages for 6 days in the presence of macrophage colony-stimulating factor (M-CSF), and on day 7, they were stimulated with IFN-γ and lipopolysaccharide (LPS) for 24 h to generate M1 macrophages. We either mock treated these or exposed them to live or UV-inactivated HCMV as described above. As shown by the results in Fig. 1C, M macrophages treated with live or UV-inactivated virus showed higher levels of secreted IL-1β than uninfected cells. Interestingly, UV-HCMV treatment resulted in higher levels of secreted IL-1β than were seen in live-HCMV-infected samples, corroborating what was observed for THP-1 cells (Fig. 1A). Collectively, these results suggest that differentiated myeloid cells process and secrete the IL-1β cytokine in response to HCMV exposure and that either replicationally inactive virus particles represent a more powerful innate inducing stimulus or live virus encodes phenotypes that dampen the processing or release of the cytokine.

### HCMV-induced IL-1β release requires the AIM2/ASC/caspase-1 inflammasome.

**FIG 2:**
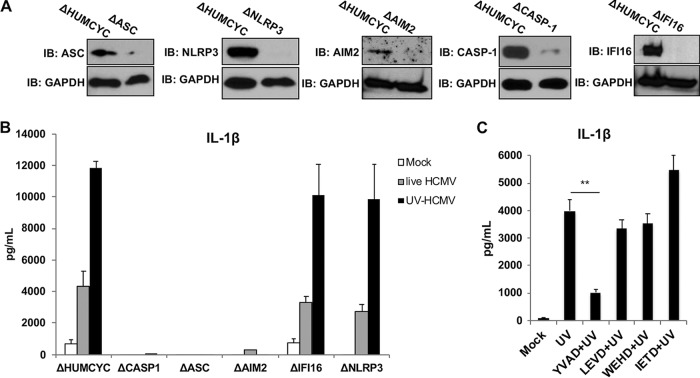
Secretion of IL-1β in response to live and UV-inactivated HCMV requires the AIM2 inflammasome. (A) Immunoblot assay for caspase-1, ASC, NLRP3, IFI16, AIM2, and GAPDH in THP-1 cells transduced with Cas9 and guide RNA (gRNA) targeting HUMCYC pseudogene or in indicated CRISPR-generated knockout cells. (B) IL-1β secretion in THP-1 cells with the HUMCYC pseudogene or gene encoding an indicated protein deleted was measured following mock treatment (white) or treatment with live (gray) or UV-inactivated (black) HCMV (HCMV-TB40/E; MOI of 3) for 18 h. Values are averages ± SEM from three biological replicates. (C) IL-1β secretion in differentiated THP-1 cells following mock treatment or treatment with UV-HCMV (UV) alone or in combination with caspase-1 inhibitor (YVAD), caspase-4 inhibitor (LEVD), caspase-5 inhibitor (WEHD), or caspase-8 inhibitor (IETD) for 18 h. Values are averages ± SEM from three replicates. One-way ANOVA with Bonferroni correction was performed. **, *P* < 0.01.

Canonical processing and secretion of IL-1β involves ASC-mediated activation of caspase-1, which targets pro-IL-1β for proteolytic cleavage. However, instances of caspase-1-independent IL-1β are known that, in humans, involve caspase-4, -5, and -8 (53–55). Based on this, we next examined whether the IL-1β processing we observed following exposure to HCMV occurs in a manner that requires the caspase-1 and ASC inflammasome. We therefore used CRISPR-Cas9-mediated genome editing to construct THP-1 cells that lack ASC or caspase-1, as shown by the results in Fig. 2A. Cells stably transduced with CRISPR components targeting a human pseudogene (HUMCYCPS3) were used to control for any potential anomalous effects caused by constitutive overexpression of the Cas9 enzyme or guide RNA (gRNA). We next examined the secretion of IL-1β from these cells following mock and virus treatments as described above. As shown by the results in Fig. 2, CRISPR-Cas9 targeting a pseudogene exhibited no measurable effect on IL-1β secretion in response to HCMV particles. However, cells lacking ASC and caspase-1 failed to secrete detectable cytokine in response to either live or UV-HCMV. These results indicate that these components of the canonical inflammasome are required for HCMV-associated IL-1β secretion.

Next, we explored the potential involvement of inflammasome-specific PRRs in this response. AIM2, IFI16, and NLRP3 have each been identified as playing essential roles in activation of the caspase-1 inflammasome by herpesvirus species (9, 10, 41). We therefore constructed THP-1 cells lacking each of these proteins (Fig. 2A) that were then differentiated and exposed to virus as described above, and IL-1β secretion measured by enzyme-linked immunosorbent assay (ELISA). Cell lines lacking IFI16 or NLRP3 responded to treatment with live or UV-HCMV in a manner highly similar to the response of control cells (Fig. 2B). However, IL-1β secretion was absent from cells lacking AIM2, suggesting that this receptor is required for inflammasome activation by the virus, an observation consistent with what has been shown for murine cytomegalovirus (56).

To examine whether other caspase proteins may contribute to or be necessary for the secretion of IL-1β in response to HCMV, we utilized small molecule inhibitors specific for caspase-1, -4, -5, and -8 (57). As shown by the results in Fig. 2C, only inhibition of caspase-1 led to a significant decrease in IL-1β levels following exposure to UV-HCMV. This finding is consistent with caspase-1 representing the sole proteolytic factor responsible for processing and release of mature IL-1β and agrees with reverse genetics loss-of-function assays (Fig. 2B).

### The cGAS-STING-dependent type I IFN response contributes positively to HCMV-induced IL-1β secretion.

**FIG 3:**
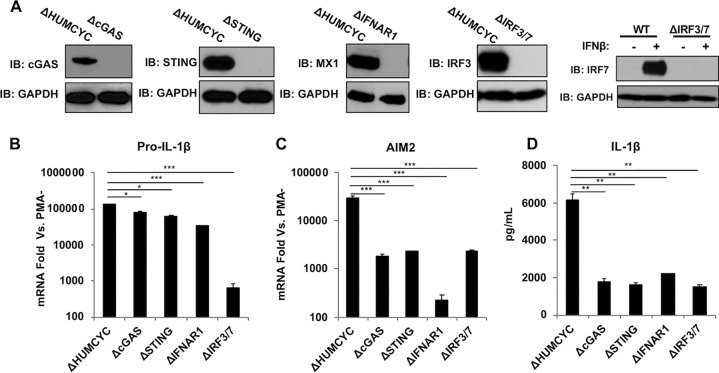
The cGAS-STING-IFN pathway contributes to IL-1β secretion in response to UV-inactivated HCMV. (A) Immunoblot assay for cGAS, STING, IFNAR1, IRF3/IRF7, and GAPDH in THP-1 cells transduced with Cas9 and gRNA targeting HUMCYC pseudogene or in indicated CRISPR-generated knockout cells. WT, wild type. (B, C) Pro-IL-1β (B) and AIM2 (C) mRNA levels in THP-1 cells transduced with Cas9 and gRNA targeting the HUMCYCPS3 (ΔHUMCYC) pseudogene or indicated cellular gene(s) following treatment with UV-inactivated HCMV-TB40/E (MOI of 3) for 18 h. Transcript fold changes are expressed as relative to the levels in untreated cells (PMA−). (D) IL-1β secretion in THP-1 cells lacking indicated protein(s) following exposure to UV-inactivated HCMV-TB40/E (MOI of 3) for 18 h. Values are averages ± SEM from three biological replicates. One-way ANOVA with Bonferroni correction was performed. *, *P* < 0.05; **, *P* < 0.01; ***, *P* < 0.001.

Previous work revealed that the type I IFN and inflammasome responses function in a complicated coregulatory relationship (reviewed in reference 58). For example, IFN both upregulates key inflammasome components like AIM2 (7) and yet also inhibits inflammasome activity (59). Moreover, activated caspase-1 cleaves the cytoplasmic dsDNA PRR cGAS, thereby inhibiting subsequent IFN induction by way of signaling through STING, IRF3 and IRF7 (IRF3/-7), and IFN (60). In contrast, signaling via IRF3/-7 and type I IFN may also induce the expression of functional inflammasome components. Importantly, HCMV infection of THP-1 cells is known to trigger IRF3-mediated IFN responses in a cGAS/STING-dependent manner (39). We therefore examined the importance of HCMV-mediated IFN induction and subsequent autocrine/paracrine signaling via the IFN receptor (IFNAR) in inflammasome activity. For this, we constructed THP-1 cells lacking cGAS, STING, IFNAR1, or IRF3 and IRF7 using CRISPR-Cas9 (Fig. 3A). As shown by the results in Fig. 3B, synthesis of pro-IL-1β mRNA as measured by quantitative PCR (qPCR) was induced in PMA-differentiated THP-1 cells relative to that in untreated cells following exposure to UV-HCMV. However, pro-IL-1β upregulation in the absence of cGAS, STING, or IFNAR1 was significantly reduced relative to its level in control cells (Fig. 3B). Surprisingly, deletion of IRF3/-7 led to the largest reduction in UV-HCMV-induced transcription. These results suggest that the functional effects of IRF3/-7 proteins in this phenomenon may be distinct from (or independent of) downstream IFN-mediated processes. We also examined UV-HCMV-induced expression of the essential PRR AIM2 in these cells. As expected, cells lacking IFN-dependent JAK/STAT signaling due to the absence of IFNAR1 exhibited the largest reduction in AIM2 upregulation (Fig. 3C) (61, 62). Cells lacking cGAS, STING, and IRF3/-7 also displayed significantly reduced levels of AIM2 induction relative to that in control cells, although to a lesser degree than following IFNAR1 deletion (Fig. 3B). These results suggest an important but not essential role for the cGAS-STING-IRF3 signaling axis in HCMV-associated AIM2 induction and that the IFN-dependent (JAK/STAT) pathway is more significant with regard to this process. We next asked whether the secretion of IL-1β was affected by the absence of these proteins. As shown by the results in Fig. 3D, in cells lacking cGAS, STING, IFNAR1, or IRF3/-7, secretion of IL-1β in response to UV-HCMV was significantly reduced but not eliminated. Collectively, these results suggest that the activation of the cGAS-STING-IRF3 signaling cascade and subsequent IFN-dependent JAK/STAT pathway by HCMV strongly contributes to but is not wholly required for IL-1β secretion. It is likely that additional innate signaling molecules, such as PRRs and transcription factors shown to be responsive to HCMV infection, including ZBP1 (40), RIG-I (63), or IRF1 (28, 64), play important contributory roles that require further investigation to mechanistically understand this in greater detail.

### Live HCMV inhibits secretion of IL-1β in UV-HCMV-treated cells.

**FIG 4:**
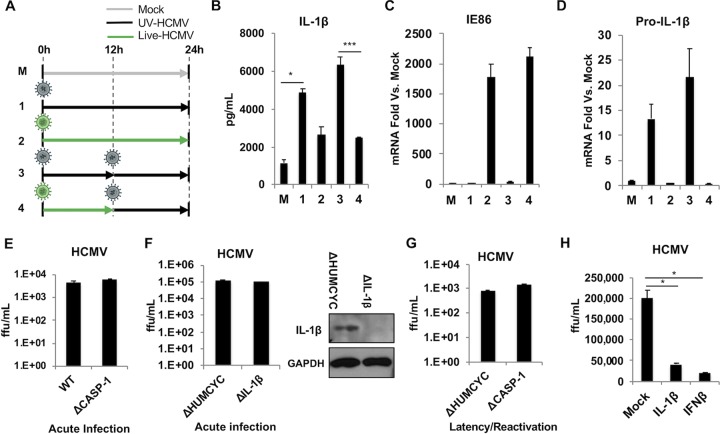
Infection of THP-1 cells with live HCMV inhibits IL-1β secretion in response to UV-HCMV. (A) Schematic depicting five experimental treatments of differentiated THP-1 cells, including mock treatment (condition M), UV-inactivated HCMV for 24 h (condition 1), live HCMV for 24 h (condition 2), UV-inactivated HCMV for 12 h and then retreatment with UV-inactivated virus for 12 h (condition 3), or live HCMV for 12 h and then UV-HCMV virus for 12 h (condition 4). (B) IL-1β secretion measured by ELISA following indicated treatments. (C, D) mRNA levels for IE86 (C) and pro-IL-1β (D) measured by qPCR following indicated treatments. Transcript fold changes are expressed as relative to levels in untreated cells (mock). Values are averages ± SEM from three biological replicates. One-way ANOVA with Bonferroni correction was performed. *, *P* < 0.05; ***, *P* < 0.001. (E) Differentiated wild-type (WT) or ΔCASP-1 THP-1 cells were infected with HCMV-TB40/E (MOI of 3) for 6 days, and titers in supernatants from these cells were determined on human fibroblasts. Values are averages ± SEM from three biological replicates. ffu, focus-forming units. (F) Differentiated ΔHUMCYC or ΔIL-1β THP-1 cells were infected with HCMV-TB40/E (MOI of 3) for 6 days, and titers in supernatants from these cells were determined on human fibroblasts. Values are averages ± SEM from three biological replicates. Immunoblot assay shows successful knockout of IL-1β in ΔIL-1β cells. (G) WT or ΔCASP-1 THP-1 cells were infected with HCMV-TB40/E (MOI of 10) for 5 days and then treated with PMA to induce HCMV reactivation. Supernatants were collected 7 days post-PMA treatment, and titers determined on human fibroblasts. Values are averages ± SEM from three biological replicates. (H) Telomerized human fibroblasts (THF) were treated with 2 ng/ml of recombinant IL-1β or with 1,000 U of IFN-β overnight and then infected with HCMV-TB40/E (MOI of 3) for 5 days. Titers in supernatants from THF were next determined on human fibroblasts. Values are averages ± SEM from three biological replicates. One-way ANOVA with Bonferroni correction was performed. *, *P* < 0.05.

As illustrated by the results in Fig. 1 and 2, infection of THP-1 cells with live HCMV resulted in significantly lower levels of IL-1β processing and secretion than did treatment with UV-inactivated virus. Based on this observation, we hypothesized that the process of infection triggers an inflammasome-mediated response but that HCMV-encoded factors expressed by live virus act to inhibit this, resulting in lower secretion of IL-1β. It is alternatively possible that live but not UV-HCMV could enter the host cell in a manner that triggers innate activation in a qualitatively different manner (e.g., with lower efficiency or potency). It is worth noting that while longer PMA differentiation was found to increase the efficiency of HCMV infectivity, this had no disproportionate impact on the inhibition of IL-1β by the virus. To investigate these possibilities, we examined IL-1β processing and secretion by UV-HCMV-treated cells that were preinfected with live HCMV. A schematic of the experimental conditions is presented in Fig. 4A. As shown by the results in Fig. 4B, THP-1 cells treated with UV-HCMV for 24 h (24 h UV [condition 1]) secrete high levels of IL-1β compared to its secretion by mock-treated cells, while cells treated with live HCMV for 24 h (24 h live [condition 2]) show significantly lower levels, as expected. Cells that were treated for 12 h with UV-HCMV and then retreated with UV-HCMV for an additional 12 h (12 h UV→12 h UV [condition 3]) secrete levels of IL-1β comparable to those seen with 24-h UV-HCMV treatment. Intriguingly, cells first infected with live HCMV for 12 h and then exposed to UV-HCMV for 12 h (12 h live→12 h UV [condition 4]) show significantly lower levels of IL-1β secretion, mimicking the levels in cells exposed to live virus for 24 h. These results indicate that prior infection with live virus impairs the striking induction of IL-1β by UV-inactivated virus. As shown by the results in Fig. 4C, synthesis of viral IE86 mRNA was only detectable when cells were exposed to live HCMV and not when they were exposed to UV-inactivated HCMV, an additional confirmation that these particles are not replication competent.

We next began to investigate the mechanistic basis of this inhibitory phenomenon associated with live HCMV by measuring the transcription of the pro-IL-1β gene. Synthesis of IL-1β mRNA has been linked with the transcription factor NF-κB (65–67). Interestingly, previous work has demonstrated inhibition of NF-κB-dependent transcription during infection with HCMV (reviewed in reference 68). We therefore examined whether the reduced IL-1β secretion observed in the presence of live virus was associated with significantly reduced levels of IL-1β mRNA. For this, we performed qPCR on cells treated as shown in Fig. 4A. Interestingly, the results in Fig. 4D illustrate that treatment with UV-HCMV alone was associated with a strong induction of pro-IL-1β transcription relative to that in mock-treated control cells. However, exposure to live virus led to inhibition of IL-1β transcriptional induction and even prevented UV-HCMV-mediated upregulation of the gene. Unfortunately, examining whether live HCMV can subsequently inhibit responses triggered by UV-HCMV is complicated by the fact that UV-HCMV represents an exceptionally potent stimulus of IRF3- and IFN-mediated antiviral innate responses, rendering cells highly refractory to normal HCMV infection (28, 29, 69). Based on this, we conclude that live HCMV inhibits both the secretion of IL-1β in response to UV-HCMV exposure and virion-associated synthesis of pro-IL-1β mRNA.

Inhibition of innate intracellular processes by viruses is commonly evolutionarily selected in response to the overt antiviral effects exhibited by them. Since IL-1β secretion is impaired during live HCMV infection, we therefore asked whether any inhibitory effects on HCMV replication are detected in the presence of a functional inflammasome relative to such effects in cells in which it is not operational. For this, we examined acute growth of HCMV strain TB40E in THP-1 cells lacking caspase-1 and in control cells, yet no measurable differences in viral titers were observed between these two cell types (Fig. 4E). We also examined whether IL-1β specifically may associate with measurable antiviral effects by disrupting the pro-IL-1β coding region using CRISPR-Cas9 from THP-1 cells (Fig. 4F). However, as with inflammasome deficiency, virus grew to similar titers in these cells and control cells (Fig. 4F). Myeloid-derived cells represent the primary reservoir of latent HCMV infection, and THP-1 cells can be used to model latency and reactivation (70, 71). We therefore next asked whether inflammasome activity exerts any obstruction of this form of HCMV replication. No significant differences in viral reactivation from latency were detected between the cell types (Fig. 4G).

Finally, recently published work reveals that IL-1β can both trigger the expression of genes conventionally classified as IFN stimulated in human foreskin fibroblasts and generate in these cells a state refractory to virus replication (20). Since these cells are permissive for and commonly used to investigate HCMV, we asked whether exposure to recombinant IL-1β rendered them inhibitory to HCMV growth. As shown by the results in Fig. 4H, the titers of HCMV grown on human foreskin fibroblasts pretreated with IL-1β were significantly lower than the titers on control cells. While the decrease in titer on these cells was not multilog, it is interesting to note that control cells pretreated with IFN-β displayed a roughly similar degree of inhibition of HCMV growth. Overall, since no differences in acute replication or reactivation from latency are observed between control THP-1 cells and those from which key inflammasome components are deleted, we conclude that this response exhibits no overt inhibitory effect on HCMV growth in these cells. However, a statistically significant drop in titers was observed in human fibroblasts pretreated with IL-1β, the degree of which was similar to that induced by pretreatment with IFN-β. Based on this, it is possible that inhibition of IL-1β may confer direct proviral effects on nonmyeloid cells.

### HCMV immediate early protein IE86 inhibits UV-HCMV-induced pro-IL-1β mRNA transcription and mature protein secretion.

**FIG 5:**
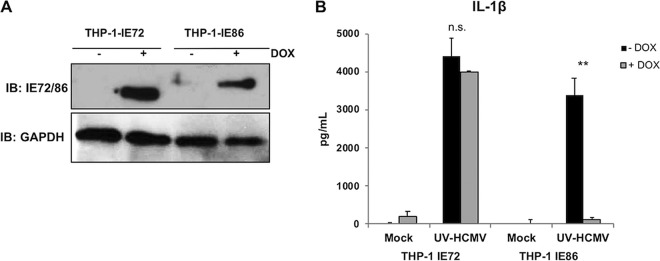
UV-HCMV-induced IL-1β secretion is blocked in the presence of HCMV IE86 but not IE72 protein. (A) Immunoblot assay showing results for IE72, IE86, and GAPDH in THP-1 cells ectopically expressing IE72 (THP-1-IE72) or IE86 (THP-1-IE86) under the control of a doxycycline (DOX)-inducible promoter in the presence but not absence of DOX. (B) IL-1β secretion from THP-1-IE72 and THP-1-IE86 cells left untreated (mock) or following exposure to UV-inactivated HCMV-TB40/E (MOI of 3) for 18 h in the presence (gray) or absence (black) of DOX. Values are averages ± SEM from three biological replicates. One-way ANOVA with Bonferroni correction was performed. n.s., not significant; **, *P* < 0.01.

The observations presented in Fig. 1 and 4 indicate that live but not UV-inactivated HCMV is associated with decreased virion-induced IL-1β processing and secretion, as well as pro-IL-1β transcription. Moreover, since inhibition was evident by 12 h postinfection, we predicted that a viral gene product synthesized soon after virus-cell contact might be responsible for these effects. HCMV contains at least five mRNAs that are expressed immediately upon cell entry and prior to the expression of other viral genes (reviewed in reference 72). The most abundant transcripts encode immediate early proteins of 72 kDa (immediate early 1 [IE1] or IE72) and 86 kDa (IE2 or IE86). Importantly, IE86 is known to inhibit NF-κB-dependent transcription by directly preventing binding of the transcription factor to promoter DNA (but not its activation or nuclear translocation), thus providing an additional mechanistic rationale for investigating these proteins (49, 73). We therefore asked whether either was sufficient to block IL-1β secretion in response to UV-HCMV exposure. To address this, we used lentivectors to construct THP-1 cell lines that express IE72 or IE86 under the control of a stably transfected doxycycline (DOX)-responsive transcriptional activator (termed THP-1-IE72 and THP-1-IE86, respectively). Efficient DOX-dependent expression of the respective proteins in these cells is illustrated in Fig. 5A. We next tested whether the presence of either IE protein impacted IL-1β secretion in response to UV-HCMV exposure. After PMA differentiation, THP-1-IE72 and THP-1-IE86 cells were treated with DOX overnight to activate protein expression and then exposed to UV-inactivated HCMV for 18 h. The results in Fig. 5B show that in both the presence and absence of DOX, treatment with UV-HCMV induced substantial IL-1β secretion in THP-1-IE72 cells, indicating that ectopic gene expression as a molecular process in this platform confers no measurable impact on the response. In THP-1-IE86 cells, UV-HCMV treatment also triggered IL-1β secretion in the absence of DOX. However, when cells were treated with DOX, secretion of the cytokine in response to UV-HCMV was eliminated. These results suggest that the IE86 but not the IE72 protein is sufficient to block HCMV-triggered IL-1β secretion.

### HCMV IE86 does not affect inflammasome function or caspase-1 activity.

**FIG 6:**
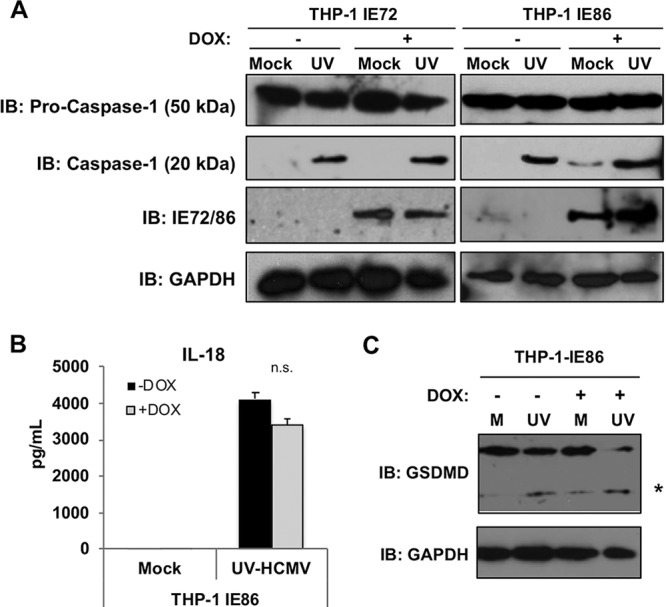
HCMV IE72 and IE86 do not inhibit UV-HCMV-induced caspase-1 cleavage, IL-18 secretion, or GSDMD cleavage. (A) Immunoblot assay for pro-IL-1β, pro-caspase-1, active caspase-1, IE72/IE86, and GAPDH in THP-1-IE72 and THP-1-IE86 cells either left untreated (mock) or treated with UV-inactivated HCMV-TB40/E (MOI of 3) for 18 h in the presence or absence of doxycycline. (B) IL-18 secretion in THP-1-IE86 cells left untreated (mock) or infected with UV-inactivated HCMV-TB40/E (MOI of 3) for 18 h in the presence (gray) or absence (black) of doxycycline. Values are averages ± SEM from three biological replicates. One-way ANOVA with Bonferroni correction was performed. n.s., not significant. (C) Immunoblot assay for total and cleaved (*) GSDMD and GAPDH in THP-1-IE86 cells either untreated (M) or infected with UV-inactivated (UV) HCMV-TB40/E (MOI of 3) for 18 h in the absence (−) or presence (+) of doxycycline.

The results presented above demonstrate that live HCMV is capable of inhibiting transcription of pro-IL-1β mRNA (Fig. 4C) and that expression of the IE86 protein alone associates with strongly diminished secretion of the mature cytokine in response to UV-HCMV exposure (Fig. 5B). To understand the latter observation, we decided to pursue whether evidence exists that impairment of inflammasome function is associated with IE86 expression. For this, we used the THP-1-IE72 and THP-1-IE86 cell lines described above (Fig. 5). We first examined whether any overt effects on caspase-1 were detectable by measuring the protein’s abundance and UV-HCMV-induced maturation in the presence of IE86 or IE72. As shown by the results in Fig. 6A, treatment of THP-1-IE72 cells with UV-HCMV led to no substantial changes in overall levels of the immature 50-kDa pro-caspase-1 protein. Also, the UV-HCMV-induced maturation of caspase-1 into its active 17-kDa form appeared normal in both the presence and absence of IE72. Likewise, the overall abundance and UV-HCMV-associated processing of caspase-1 when IE86 is expressed also appeared unchanged, suggesting that inflammasome function is not an inhibitory target for the protein. While caspase-1 appears to mature normally in the presence of IE86, it is still possible that proteolytic activity of the mature protein is impaired, and this could explain decreased IL-1β secretion. Since we show above that HCMV induces IL-1β secretion by way of the canonical AIM2/ASC/caspase-1 inflammasome (Fig. 2), any inhibition of the proteolytic abilities exhibited by this complex would likely also result in diminished secretion of IL-18, as well as impaired processing of the gasdermin-D (GSDMD) protein. To establish whether caspase-1 activity is affected by IE86, we therefore examined the maturation of IL-18 and GSDMD as alternative caspase-1 proteolytic targets. As shown by the results in Fig. 6B, IE86 exhibited no significant inhibitory effect on the secretion of IL-18 occurring in response to UV-HCMV exposure. Consistent with this result, cleavage of GSDMD also occurred normally in the presence of IE86 (Fig. 6C). Based on these combined results, we conclude that IE86 does not lead to diminished IL-1β secretion via a phenotype that prevents caspase-1 maturation or proteolytic activity.

### IE86 is associated with diminished levels of both endogenous and constitutively expressed transgenic pro-IL-1β protein.

**FIG 7:**
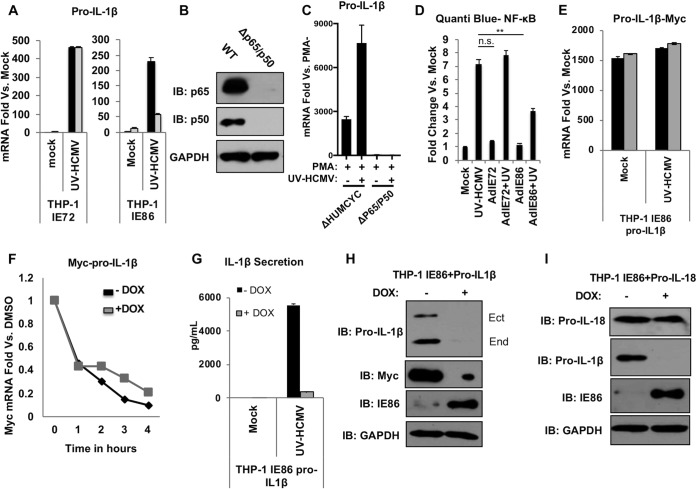
HCMV IE86 impairs transcription of endogenous but not constitutively expressed ectopic pro-IL-1β. (A) Pro-IL-1β mRNA levels in THP-1-IE72 or THP-1-IE86 cells left untreated (mock) or infected with UV-inactivated HCMV-TB40/E (MOI of 3) for 18 h in the presence (gray) or absence (black) of doxycycline. (B) Immunoblot assay for NF-κB subunits P65 and P50, as well as GAPDH, from parental THP-1 cells or those from which the indicated proteins were deleted using CRISPR-Cas9. (C) Pro-IL-1β mRNA levels in control (ΔHUMCYC) THP-1 cells or those from which P65/P50 have been deleted, as indicated, following treatment with PMA or UV-HCMV (MOI of 3). Values presented are mean mRNA fold changes ± SEM calculated relative to levels in control cells not treated with PMA (PMA−). (D) THP-1 NF-κB Quanti-Blue reporter cells were transduced with recombinant adenovirus expressing IE72 or IE86 (AdIE72 and AdIE86) overnight and then mock treated or treated with UV-HCMV (MOI of 3) for an additional 18 h. Values are averages ± SEM from three replicates. One-way ANOVA with Bonferroni correction was performed. n.s., not significant; **, *P* < 0.01. (E) Levels of Myc-specific mRNA in THP-1-IE86 cells ectopically expressing Myc-tagged pro-IL-1β that were either left untreated (mock) or infected with UV-inactivated HCMV-TB40/E (MOI of 3) for 18 h in the presence (gray) or absence (black) of doxycycline. Data are expressed as average levels of transcript ± SEM relative to levels in untreated cells (mock) from three biological replicates. (F) Constitutive ectopic pro-IL-1β mRNA degradation was examined by quantifying transcript levels at indicated times after the addition of actinomycin D using qPCR in the presence (gray) or absence (black) of IE86. Transcript levels are expressed relative to levels in DMSO-treated cells. (G) IL-1β secretion in THP-1-IE86 cells ectopically expressing Myc-tagged pro-IL-1β that were either left untreated (mock) or infected with UV-inactivated HCMV (MOI of 3) for 18 h in the presence (gray) or absence (black) of doxycycline. (H) Immunoblot assay for pro-IL-1β (ectopic and endogenous, as indicated), Myc (ectopic pro-IL-1β), IE86, and GAPDH in THP-1-IE86 cells ectopically expressing Myc-tagged pro-IL-1β under a constitutive promoter. (I) Immunoblot assay for pro-IL-18, pro-IL-1β, IE86, and GAPDH in THP-1-IE86 cells ectopically expressing pro-IL-18 under a constitutive promoter.

Inhibition of IL-1β secretion but not inflammasome or caspase-1 function by IE86 is consistent with but not demonstrative of the protein’s described phenotype that involves preventing NF-κB binding to promoter regions (49, 73). We therefore examined whether induced synthesis of endogenous pro-IL-1β mRNA was inhibited in the presence of IE86. As shown by the results in Fig. 7A, the levels of UV-HCMV-activated pro-IL-1β mRNA are greatly diminished during DOX-induced ectopic protein expression in THP-1-IE86 but not THP-1-IE72 cells. This implied that the IE86-associated reduction in IL-1β secretion could be dependent upon the protein’s ability to impair pro-IL-1β mRNA synthesis, likely via its targeting of NF-κB. We next verified the essentiality of NF-κB for HCMV-mediated pro-IL-1β transcription by first constructing THP-1 cells that lack the P50 and P65 transcription factor subunits, as shown by the results in Fig. 7B. We then used qPCR to measure pro-IL-1β mRNA induction in these cells following treatment with either PMA or PMA plus UV-HCMV. As shown by the results in Fig. 7C, the levels of pro-IL-1β mRNA are strongly induced by both stimuli in control cells and yet are undetectable in cells lacking P50 and P65. These results confirm the requisite role of NF-κB in transcriptional activation of pro-IL-1β.

To additionally verify the ability of IE86 to block NF-κB-dependent transcription, we utilized a THP-1 reporter cell line that expresses a secreted alkaline phosphatase reporter protein in response to NF-κB-mediated activity (Quanti-Blue THP-1). We used recombinant adenovectors to express either IE72 or IE86 in these cells as described previously (74) and then exposed them to UV-HCMV. As illustrated by the results in Fig. 7D, the NF-κB-dependent reporter signal was strongly stimulated by UV-HCMV treatment and unaffected by the presence of IE72. In contrast, IE86 led to a significant decrease in the induction of reporter expression by UV-HCMV. This result is consistent with previous work showing that IE86 blocks the expression of NF-κB-dependent genes by preventing the formation of an NF-κB transcriptional protein complex on genomic promoter regions (49).

We next decided to investigate the role of IE86-mediated reduction in IL-1β mRNA on IL-1β secretion by constructing a THP-1-IE86 cell line that constitutively expresses a Myc-tagged version of pro-IL-1β under the constitutive human EF1α promoter. This approach eliminates NF-κB from the transcriptional control of pro-IL-1β levels and, crucially, any impact of the IE86-mediated inhibitory phenotype that results in diminishment of pro-IL-1β mRNA. As shown by the results in Fig. 7E, synthesis of the Myc-containing pro-IL-1β mRNA was unchanged following DOX-dependent expression of IE86 in either the presence or absence of UV-HCMV treatment, indicating that transcription occurs with uniformity in this model. We also examined whether IE86 associates with changes in pro-IL-1β mRNA stability. For this, we exposed THP-1-IE86-pro-IL-1β cells to DOX or left them untreated as described in the legend to Fig. 7E. We then treated the cells with actinomycin D to terminate all mRNA synthesis, harvested total RNA after 1 h, 2 h, 3 h, or 4 h, and performed qPCR to compare the respective levels of pro-IL-1β mRNA. No significant differences were observed in the presence or absence of DOX-induced IE86, suggesting that the protein does not exhibit an effect on the half-life of pro-IL-1β mRNA (Fig. 7F).

We next asked whether ectopic IE86 conferred an impact on the secretion of IL-1β when it was unable to affect the levels of the corresponding mRNA. Remarkably, the expression of IE86 was found to associate with a nearly complete block in IL-1β secretion following treatment with UV-HCMV, as illustrated by the results in Fig. 7G. Since the protein is unable to influence pro-IL-1β mRNA abundance in these cells (Fig. 7E and F) and does not block caspase-1 activity (Fig. 6), this observation suggests that IE86 prevents IL-1β secretion via an alternative activity. We next examined whether IE86 was associated with observable changes in the levels of endogenous or transgenic IL-1β protein. As shown by the results in Fig. 7H, inducible expression of IE86 led to the elimination of both endogenous and ectopic pro-IL-1β protein. To determine whether the expression of IE86 associates with degradation of other proteins expressed in this manner, we used identical methods to construct THP-1 cells that constitutively express pro-IL-18 as well as DOX-inducible IE86. The results in Fig. 7I illustrate that IE86 does not affect the levels of ectopic pro-IL-18 in these cells, suggesting that the effect displays specificity with respect to target protein. Collectively, these results indicate that IE86 exhibits at least two distinct phenotypes directed at the IL-1β response. First, the protein inhibits the expression of endogenous pro-IL-1β mRNA, likely via its impairment of NF-κB activity (49, 73). Second, IE86 is also associated with degradation of the pro-IL-1β protein.

### Levels of constitutively expressed pro-IL-1β protein are reduced in HCMV-infected cells.

**FIG 8:**
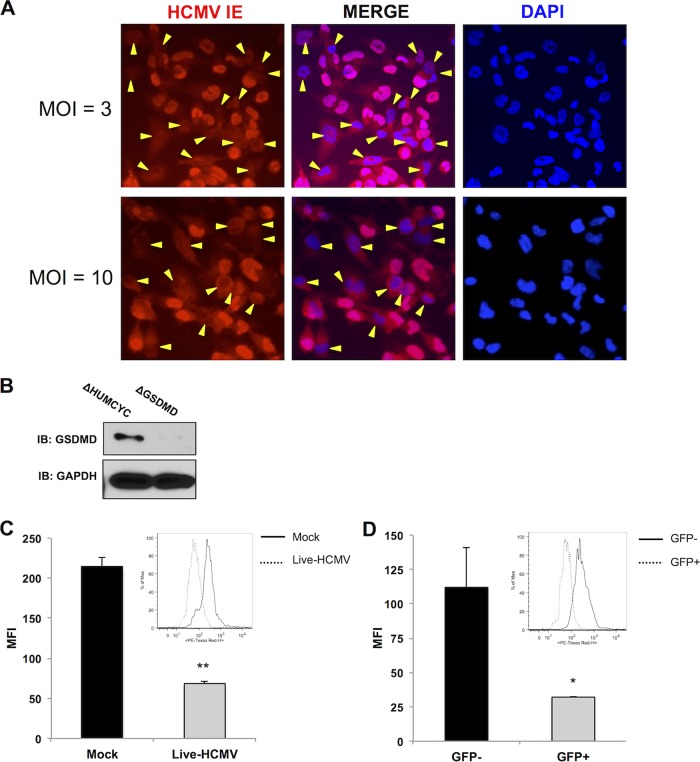
Levels of pro-IL-1β are reduced in HCMV-positive cells. (A) Immunofluorescence microscopy showing HCMV IE expression in THP-1 cells infected at the indicated MOI overnight. Yellow markers indicate DAPI-stained cells that lack detectable nuclear IE protein. (B) Immunoblot assay for GSDMD in ΔHUMCYC and ΔGSDMD THP-1 cells. (C, D) Intracellular staining for IL-1β in ΔGSDMD EF1α-pro-IL-1β THP-1 cells either mock infected or infected with HCMV-TB40/E (MOI of 3) for 18 h (C) and gated for virally encoded GFP (D). GFP-positive (GFP+) population depicts the number of HCMV-infected cells in the sample. The bar graphs show the mean fluorescence intensities (MFI) for IL-1β staining. The insets show IL-1β staining in the indicated populations. Values are averages ± SEM from two biological replicates. Student’s *t* test was performed. *, *P* < 0.05; **, *P* < 0.01.

The results described thus far indicate that UV-inactivated HCMV is capable of triggering high levels of IL-1β secretion from monocytic cells (Fig. 1 and 2) but that live HCMV (Fig. 1, 2, and 4) and IE86 alone (Fig. 5 and 7) are capable of drastically reducing this. Moreover, IE86 in isolation is also able to inhibit the transcription of endogenous pro-IL-1β and eliminate both secreted IL-1β and the immature pro-IL-1β protein (Fig. 7). However, the data presented in Fig. 1B indicate that the decrease in processed and secreted IL-1β associated with live-virus infection is not accompanied by a concomitant reduction in the levels of pro-IL-1β. This apparent inconsistency may be due to technical conditions, such as the inherently low dynamic range of immunoblot exposure that renders comparative quantification of protein levels difficult. It may also relate to the inefficiency of acute THP-1 cell infection by HCMV that results in a proportionally low percentage of IE protein-expressing cells when a high multiplicity of infection (MOI) is used, due to abortive infection after virion entry. This is illustrated by indirect immunofluorescence, as shown in Fig. 8A. In this case, degradation phenotypes directed at pro-IL-1β may be difficult to detect since protein levels from a large portion of uninfected but virus-exposed cells may overwhelm virus-associated reductions.

To overcome these experimental barriers, we aimed to determine whether diminution of pro-IL-1β protein was detectable specifically in cells actively infected with HCMV. Unfortunately, since IE86 is required for viral growth, using a reverse genetics approach to demonstrate the protein’s essentiality for this phenotype by constructing a deletion mutant is not feasible. We therefore decided to first use CRISPR-Cas9 to delete GSDMD from the THP-1-pro-IL-1β cells described above (Fig. 8B). Since mature IL-1β is secreted through GSDMD pores formed in response to inflammasome activation (75), we surmised that cells lacking the protein would accumulate both mature and immature IL-1β intracellularly. Infection of these cells with HCMV expressing a green fluorescent protein (GFP) reporter protein would then allow quantification of constitutively expressed Myc-tagged pro-IL-1β in infected versus uninfected cells using flow cytometry. As shown by the results in Fig. 8C, the intracellular levels of Myc-containing protein are significantly lower in HCMV-infected cells than in separately treated mock-infected control cells. Moreover, when we focused on the HCMV-infected and uninfected cells from the same culture, we observed that intracellular levels of Myc-tagged protein were lower in GFP-positive than in GFP-negative cells in the experiment whose results are shown in Fig. 8D. Interestingly, a subset of infected cells exhibited pro-IL-1β protein levels that overlapped in abundance with the level observed in uninfected cells. This may be due to functionally important interactions with host or viral factors that are transient or deviate kinetically from the timing of our measurements. Overall, these results indicate that HCMV infection leads to a decrease in intracellular IL-1β in a manner that is both independent of a viral block to pro-IL-1β transcription and consistent with our other observations illustrating virus- and IE86-mediated reduction in IL-1β secretion.

## DISCUSSION

Previous characterization of the innate immune response to infection with HCMV has described important roles for IRF3-terminal, dsDNA-specific PRRs (35, 37–40). Additionally, IL-1β secretion from endothelial cells, smooth muscle cells, and primary monocytes in response to HCMV infection has also been reported (31, 34, 76), in agreement with our results. However, while inflammasome activation leading to secretion of IL-1β and IL-18 can occur in response to cytoplasmic dsDNA (56, 77), multiple PRRs, including NLRP3 (9, 78, 79), IFI16 (8–10, 78, 80), and AIM2 (41, 81–84), have been implicated in herpesvirus-mediated induction of this process. Importantly, in many cases, more than one of these PRRs has been shown to be simultaneously involved in inflammasome activation by herpesvirus species (9, 81–84). To explore the respective essentiality of the primary inflammasome-activating PRRs, as well as ASC and caspase-1, we leveraged CRISPR-Cas9 technology to construct cells lacking proteins of functional interest. Genomic deletion as a technique allows loss-of-function assays to be performed that lack the ambiguity that may emerge as a result of residual gene expression seen during RNA interference-based methods. As such, it represents a more rigorous experimental platform to investigate the phenotypes of nonessential proteins. Using this approach, we determined that ASC and caspase-1 are required for HCMV-mediated IL-1β secretion, thereby functionally implicating the canonical inflammasome. Furthermore, we also found that AIM2 but neither IFI16 nor NLRP3 (other PRRs with described roles in herpesvirus-mediated inflammasome activation) is essential to this reaction.

Processes driven by type I IFN, the inflammasome, and IL-1β exist in complicated coregulatory relationships (58). Type I IFN signaling can augment inflammasome responses by being required both for bacterium-associated activation and for driving AIM2 expression (61, 62). However, IFN has also been shown to inhibit NLRP3 inflammasome activity and to suppress pro-IL-1β synthesis (59, 85, 86). Moreover, caspase-1 activation in response to DNA virus infection leads to cleavage of cGAS and a resulting block against IFN induction (60). Interestingly, cyclic dinucleotides are also capable of directly activating the inflammasome in a manner independent of dsDNA but which requires NLRP3 and AIM2 (81), and HCMV infection of myeloid cells leads to their cGAS-dependent synthesis (39, 87). Furthermore, in primary human myeloid cells, the cGAS-STING axis was found to induce lysosomal cell death in response to dsDNA, thereby leading to potassium efflux and activating NLRP3, rendering AIM2 unimportant (88). IL-1β secretion in response to UV-HCMV was significantly diminished in cells lacking cGAS or STING (Fig. 3D), a finding consistent with what has been described for cytoplasmic dsDNA in human monocytes by Gaidt et al. (88). However, IL-1β secretion was abrogated in the absence of AIM2 and unaffected by NLRP3 deletion (Fig. 2), which contrasts with the results of Gaidt et al. (88) and suggests a distinct mechanism. Since the levels of IL-1β were also diminished in cells lacking IFNAR1 and IRF3/-7, we predict that this is due to impaired transcription of functionally important genes driven by the HCMV-activated cGAS-dependent IFN pathway. AIM2 may represent the crucial node in this case, given its essentiality to HCMV-mediated IL-1β secretion (Fig. 2) and the sensitivity of its transcription to IFNAR1 deletion (Fig. 3B). Intriguingly, IRF3/-7 were found to play an important role in pro-IL-1β transcription as induced by treatment with PMA and UV-HCMV (Fig. 3B). This was unexpected, since the deletion of cGAS, STING, or IFNAR1 did not elicit the same effect, and it suggests that pro-IL-1β is perhaps a direct transcriptional target of the IRFs when activated independently of cGAS/STING or that these IRFs induce other key transcriptional activators. Overall, whether IL-1β secretion in response to HCMV was diminished rather than abrogated in the absence of IFN pathway proteins (Fig. 3E) due to functional roles for IFN-independent transcription factors in the expression of inflammasome components will require more focused investigation.

Our observations led us to hypothesize that HCMV encodes an inhibitory phenotype(s) that actively blocks IL-1β secretion. Seemingly contradictory observations have been reported regarding the effects of infection and virion inactivation by UV on IL-1β secretion from monocytes, smooth muscle cells, and endothelial cells (31, 34, 76). For instance, live HCMV has actually been reported to inhibit IL-1β secretion to below background levels in peripheral blood mononuclear cells (PBMCs) and endothelial cells (34, 89). However, while exposure of endothelial cells to UV-HCMV appears to induce higher levels of IL-1β secretion than does live virus (34), consistent with our results (Fig. 1 and 2), the opposite has been reported in monocytes (76). Our work demonstrates HCMV-associated inhibition of IL-1β secretion induced by the inactive virion as a stimulus, strongly implying that the live virus encodes a counteracting phenotype(s). As discussed below, live virus was also associated with greatly decreased pro-IL-1β transcription, suggesting this as a potential mechanism (Fig. 4D). Interestingly, while IL-1β appeared to confer no measurable antiviral effects on acute HCMV growth or reactivation from latency in THP-1 cells, a modest yet significant decrease in viral titers was observed on human fibroblasts pretreated with IL-1β (Fig. 4F and H). This is consistent with studies by Iwata et al. showing that a stromal cell line refractory to HCMV growth could be made permissive by neutralization of exogenous IL-1β (42). It is possible that the evolutionarily impactful paracrine effects conferred by IL-1β occur in stromal cells, such as fibroblasts (20), or are based on its interface with the inflammatory and adaptive immune responses, which would require *in vivo* models to investigate (21).

Since the HCMV-mediated block was both eliminated following particle inactivation and yet evident by 12 h postinfection, we hypothesized that the inhibition is conferred by an immediate early (IE) protein. The IE locus encodes three minor (55 kDa, 38 kDa, and 18 kDa) and two major (IE1 [72 kDa] and IE2 [86 kDa]) proteins. While IE72 and IE86 differ by only one exon, IL-1β secretion is blocked in the presence of ectopically expressed IE86 but not in that of IE72 (Fig. 5), strongly suggesting that the protein region(s) required for the phenotype is contained within the IE86-exclusive exon. Remarkably, IE86-mediated inhibition of IL-1β secretion was not accompanied by observable impacts on caspase-1 activation or proteolytic activity, and the protein exhibited no effect on the alternative caspase-1 targets IL-18 and GSDMD (Fig. 6). However, IE86 but not IE72 did inhibit virion-induced pro-IL-1β transcription (Fig. 7A) and the expression of an NF-κB-dependent reporter protein (Fig. 7D). This result is consistent with previous work showing that IE86 both impairs transactivation of the pro-IL-1β promoter (48) and blocks NF-κB-dependent transcription through disruption of the binding of the protein complex to promoter DNA (49). We circumvented the natural cellular processes involved in the gene’s transcription by using an artificial constitutive promoter. In cells expressing constitutive pro-IL-1β, inducible IE86 displayed no impact on mRNA synthesis or half-life (Fig. 7E and F). Astonishingly, however, IE86 still blocked virion-induced secretion of IL-1β (Fig. 7G). Collectively, these results indicate that IE86 exhibits a novel phenotype that specifically blocks virus-induced IL-1β secretion in a manner that is independent of inflammasome impairment, caspase-1 proteolytic activity, and pro-IL-1β transcription.

To understand this, we first looked at the overall levels of endogenous and transgenic constitutive pro-IL-1β protein and found that the expression of IE86 rendered both Myc-tagged and endogenous pro-IL-1β undetectable (Fig. 7H). However, cells constructed to express pro-IL-18 showed no IE86-associated decrease in this protein, indicating that this is a targeted rather than a nonspecific effect (Fig. 7I). Many phenotypes directed at innate immune responses have been described for HCMV IE proteins, including IE86 (reviewed in reference 90). IE86-mediated degradation of host immune-associated proteins, including STING (91) and CD83 (92), has even been reported. Whether IE86 employs similar biochemical mechanisms or protein domains to affect pro-IL-1β levels is an ongoing focus of investigation. Moreover, whether IE86 is also necessary for inhibitory effects seen during live-virus infection (Fig. 1, 2, and 4) is difficult to address, since the protein is required for replication and, thus, a deletion mutant is not feasible. Since IE86 is required for the expression of heterologous HCMV open reading frames (ORFs), depletion approaches like RNA interference would also make it difficult to discern the direct effects of IE86 itself from those of IE86-dependent viral (or cellular [74]) proteins. However, if IE86-inclusive amino acids (likely within exon 5) can be identified as essential for inflammasome inhibition yet nonessential for virus replication and viral gene transactivation, it may be possible to construct a viable mutant that lacks only the targeted trait. In addition, since assays like ELISA and immunoblotting utilize material collected as an aggregate (e.g., whole-cell lysates, supernatant, etc.), discerning an inhibitory phenotype in cultures that contain productively infected, abortively infected, and uninfected cells could be misleading, especially if paracrine effects are involved. We therefore aimed to examine whether the decrease in pro-IL-1β could be directly observed in actively infected cells by using GFP-expressing virus and flow cytometry. HCMV-infected (GFP-positive) cells showed significantly reduced Myc, a finding consistent with decreased IL-1β secretion and pro-IL-1β levels following HCMV infection.

**FIG 9:**
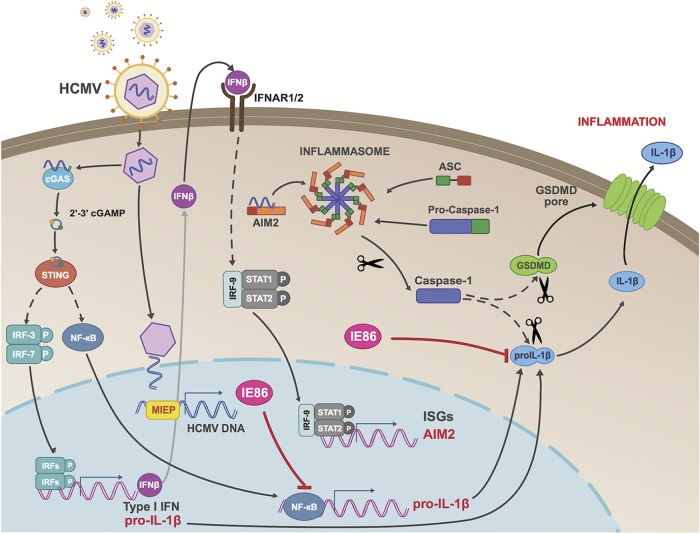
Proposed model of IFN and inflammasome activation by HCMV and IL-1β inhibition by IE86. Cellular entry of HCMV particles into THP-1 cells is detected by the dsDNA-reactive PRR cGAS, which synthesizes 2′,3′-cGAMP and triggers STING-mediated activation of IRFs and NF-κB. These then induce transcription of type I IFN and pro-IL-1β, and the IFN-dependent pathway drives AIM2 expression, enabling virus-mediated activation of the inflammasome, formation of GSDMD pores, and IL-1β secretion. Expression of the HCMV IE86 protein counteracts this response both by inhibiting pro-IL-1β transcription and by leading to degradation of the pro-IL-1β protein. MIEP, major immediate-early promoter; ISGs, interferon-stimulated genes.

In summary, we describe essential and contributory roles for dsDNA-reactive innate immune pathways, as well as the caspase-1 inflammasome, in HCMV-mediated induction of IL-1β secretion. In addition, both live virus and the IE86 protein are capable of inhibiting this process through obstruction of pro-IL-1β mRNA synthesis and degradation of the immature pro-IL-1β protein. Our proposed model for this is illustrated in Fig. 9. To our knowledge, this represents the first description of a herpesviral protein that targets multiple distinct molecular activities required for operation of the same innate immune process. Additionally, it adds a new phenotypic capacity to our understanding of IE86.

## MATERIALS AND METHODS

### Cells and virus.

THP-1 cells were obtained from ATCC and grown in RPMI 1640 complemented with 10% fetal bovine serum (FBS) and penicillin and streptomycin. THP1-Dual cells, NF-κB-SEAP (secreted alkaline phosphatase), and IRF-Lucia luciferase reporter monocytes were obtained from Invivogen and cultured as described above. THP-1 cells were differentiated in 100 ng/ml phorbol 12-myristate 13-acetate (PMA) for 24 h before stimulation unless otherwise indicated. Cell were grown at 37°C and 5% CO_2_. Titers of sucrose cushion-purified HCMV TB40/E (a gift from Felicia Goodrum [93]) expressing enhanced GFP (EGFP) were determined by 50% tissue culture infection dose (TCID_50_) assay on human fibroblasts, and the virus used to infect THP-1 cells at a multiplicity of infection (MOI) of 3. UV inactivation of HCMV was performed using the Spectrolinker XL-1000 (Spectronics Corporation) by exposing virus resuspended in 200 μl for 30 s at 600 μJ three times sequentially (69). Efficiency of virus inactivation was verified by qPCR of viral immediate early (IE) gene expression. Primary monocytes were isolated from whole blood from healthy donors in accordance with OHSU IRB protocol number 3014 and by adapting a previously described protocol based on double gradient centrifugation (94). Briefly, whole blood was first centrifuged to separate the cell population from the serum, and next, the buffy coat was overlaid on lymphocyte separation medium (1.077 g/ml; Corning) and spun to separate the peripheral blood mononuclear cells (PBMCs). PBMCs were isolated and overlaid on a Percoll (1.131 g/ml; Sigma) isosmotic solution that allowed separation of monocytes, which were then resuspended in X-Vivo 15 medium (Lonza) containing 2.5 ng/ml human M-CSF (Peprotech) and incubated for 6 days at 37°C and 5% CO_2_ for differentiation into macrophages.

### Antibodies and reagents.

The following antibiotics were used for resistance selection in lentivector-transduced cell lines: 3 μg/ml puromycin (Invivogen), 10 μg/ml blasticidin (Invivogen), 400 μg/ml neomycin (Enzo Life Sciences), and 300 μg/ml hygromycin (Enzo Life Sciences). Doxycycline (DOX) was obtained from Enzo Life Sciences and used to induce the expression of ectopically expressed proteins at 1 to 10 μg/ml. Polybrene was obtained from Sigma-Aldrich. Actinomycin D (Enzo Life Sciences) was used at 5 μg/ml to block transcription. Dimethyl sulfoxide (DMSO) was obtained from Thermo Fisher. Immunoblotting was done using antibodies to the following proteins: glyceraldehyde-3-phosphate dehydrogenase (GAPDH) (sc-51906; Santa Cruz Biotechnology), HCMV-IE86/IE72 (MAB810R; Millipore), HCMV-pp65 (clone 28-19; from Patrizia Caposio [95]), c-MYC (sc-40; Santa Cruz Biotechnology), GSDMD (sc-81868; Santa Cruz Biotechnology), IL-1β (AF-201; R&D Systems), caspase-1 (3866; Cell signaling), NLRP3 (13158; Cell Signaling), AIM2 (ab93015; Abcam), ASC (sc-22514-R; Santa Cruz Biotechnology), IFI16 (sc-8023; Santa Cruz Biotechnology), STING (13647; Cell Signaling), cGAS (15102; Cell Signaling), Mx1 (GTX111153; GenTex), IRF3 (4302; Cell Signaling), IRF7 (4920; Cell Signaling), NF-κB P65 (sc-372; Santa Cruz Biotechnology), and NF-κB P50 (sc-8414; Santa Cruz Biotechnology). Human interferon gamma (IFN-γ; PBL Assay Science) was used at 1,000 U together with 100 ng/ml LPS (Invivogen) to differentiate M0 macrophages in M1 macrophages. Human interferon beta (IFN-β; PBL assay science) was used at 1,000 U. Recombinant human IL-1β (R&D systems) was used at 2 ng/ml. Caspase-1 inhibitor carboxybenzoxy-Tyr-Val-Ala-Asp-fluoromethylketone (Z-YVAD-FMK), capase-4 inhibitor carboxybenzoxy-Leu-Glu-Val-Asp-fluoromethylketone (Z-LEVD-FMK), caspase-5 inhibitor carboxybenzoxy-Trp-Glu-His-Asp-fluoromethylketone (Z-WEHD-FMK), and caspase-8 inhibitor carboxybenzoxy-Ile-Glu-Thr-Asp-fluoromethylketone (Z-IETD-FMK) were all obtained from Enzo Life Science and used at a concentration of 2 μM. Quanti-Blue was used to quantitatively measure the activity of secreted alkaline phosphatase (SEAP; Invivogen) in THP1-Dual cells.

### Immunoblotting.

Collected cell pellets were lysed in RIPA buffer (10 mM Tris-Cl [pH 8.0], 1 mM EDTA, 1% Triton X-100, 0.1% sodium deoxycholate, 0.1% SDS, 140 mM NaCl, and protease and phosphatase inhibitor cocktail) and analyzed by SDS-PAGE. Secreted IL-1β and caspase-1 were precipitated from the supernatant as previously described (96). Briefly, 500 μl of medium from treated cells was centrifuged at 2,000 × *g* for 10 min to remove cell debris. Next, the supernatant was mixed with 500 μl of methanol and 125 μl of chloroform and vortexed. The sample was centrifuged for 5 min at room temperature at 15,000 × *g*. After centrifugation, a protein layer was visible between the aqueous and organic phases. The aqueous phase was removed without disturbing the protein layer, and next, 500 μl of methanol was added and the contents of the tube were mixed by vortexing. After another centrifugation for 5 min at 15,000 × *g*, a protein pellet was visible at the bottom of the tube. All methanol was removed from the tube, which was left open to dry for 10 min. Finally, the pellet was reconstituted with 1× loading buffer. After SDS-PAGE, proteins were transferred to polyvinylidene difluoride (PVDF) membranes using semidry transfer at 200 mA for 35 min. Blots were blocked in 5% milk (or 5% bovine serum albumin [BSA] for IE72, IE86, and STING) in 1× phosphate-buffered saline (PBS) containing 0.1% Tween 20 for 30 min at room temperature and then incubated with the appropriate primary antibodies overnight at 4°C. Next, the blots were washed three times in 1× PBS plus 0.1% Tween 20 and then incubated with horseradish peroxidase (HRP)-conjugated secondary antibodies in 5% milk for 30 min at room temperature. The blots were subsequently washed as described for the primary antibodies and visualized using enhanced chemiluminescence (Thermo Fisher).

### CRISPR-Cas9-directed genome editing.

Genome editing using lentivector-mediated delivery of CRISPR-Cas9 components was performed generally as described previously (97–99). Briefly, 20-nucleotide guide RNA (gRNA) sequences targeting protein-coding regions were inserted into the lentiCRISPRv2 vector (catalog number 52961; Addgene). These sequences were as follows: HUMCYCPS3, TAAACCTACACAACATACAC; caspase-1, GCATTGAGTTGTAGTATATC; ASC, CATGTCGCGCAGCACGTTAG; AIM2, TCTTGGGTCTCAAACGTGAA; NLRP3, GGATCTTCGCTGCGATCAAC; IFI16, TATACCAACGCTTGAAGACC; IFNAR1, AAACACTTCTTCATGGTATG; IRF3, GAGGTGACAGCCTTCTACCG; IRF7, TACACCTTGTGCGGGTCGGC; STING, CCCGTGTCCCAGGGGTCACG; cGAS, AGACTCGGTGGGATCCATCG; GSDMD, TTCCACTTCTACGATGCCA; Pro-IL-1β, CATGGCCACAACAACTGACG; NF-κB P65, GATCTCCACATAGGGGCCAG; and NF-κB P50, CAACTATGTGGGACCAGCAA.

### Transgene expression using lentivector and adenovector systems.

Myc-tagged pro-IL-1β or pro-IL-18 was cloned into and expressed from a lentiviral vector containing the constitutive EF1α promoter (pLVX*-*EF1alpha*-*IRES; Clontech). The HCMV IE72 and IE86 coding sequences were cloned into pLVX-Tight-Puro (Clontech). pLVX-Tight-Puro is a tetracycline (Tet)-inducible lentiviral expression vector designed to express a gene of interest under the control of a modified Tet-responsive promoter. Lentiviral particles were produced by transfecting a specific lentivector plasmid along with a packaging plasmid (psPAX2) (catalog number 12260; Addgene) and a vesicular stomatitis virus G protein pseudotyping plasmid (pMD2.G) (catalog number 12259; Addgene) into Lenti-X 293T cells (Clontech) using Lipofectamine-LTX (Life Technologies, Inc.). Medium was harvested at 48 h and 72 h posttransfection, centrifuged (3,000 × *g* for 10 min), and filtered through a 0.45-μm filter to remove cell debris. Subconfluent THP-1 cells were exposed to lentivirus for 8 h in the presence of Polybrene at 5 μg/ml. After the cells reached confluence, cultures were split into RPMI containing 10% FBS and 3 μg/ml puromycin. Transduced cells were passaged in the presence of puromycin for 7 to 10 days, and then protein expression/knockout was examined by immunoblotting. pAdTet7, a Tet-inducible adenoviral vector, was used to express HCMV IE72 and IE86 generally as described previously (100). Briefly, the tetracycline-responsive (Tet-On) transactivator protein is necessary in order to express proteins from either pLVX-Tight-Puro or pAdTet7. The transactivator, which is transcriptionally active in the presence of doxycycline, was encoded by the lentivector pLVX-TetON-Advanced (Clontech) and used to make stable cells following transduction. Recombinant adenoviruses were produced by cotransfection of the shuttle plasmid containing IE72 or IE86 with adenoviral DNA (Ad5-ψ5) lacking E1A/E3 sequences into HEK293T cells that express Cre recombinase (Cre4). Adenoviruses were grown using Cre4 cells, and titers determined by serial dilution on HEK293 cells.

### RNA isolation and semiquantitative reverse transcription-PCR.

Total RNA was isolated from cells, treated with DNase provided in a DNA-free RNA isolation kit (Zymo Research) according to the manufacturer’s protocol, and quantified by using UV spectrometry. Single-stranded cDNA for use as a PCR template was prepared from total RNA and random hexamers to prime first-strand synthesis via SuperScript III reverse transcriptase (Life Technologies, Inc.) according to the manufacturer’s protocol. The primer sets used for qPCR were designed using Primer-BLAST (https://www.ncbi.nlm.nih.gov), and the sequences are as follows: IE72 FWD, 5′-CGCTGGTGCTGCCAGCTCCTCTG-3′; IE72 REV, 5′-CCGCTCCTCCTGAGCACCCT-3′; IE86 FWD, 5′-ACGCACCCGCTCTCCCAGAT-3′; IE86 REV, 5′-ACGGATGCTCCTCCGCCACT-3′; IL-1β FWD, 5′-ATGGCAGAAGTACCTAAGCTCGC-3′; IL-1β REV, 5′-ACACAAATTGCATGGTGAAGTTT-3′; IL-18 FWD, 5′-TGGCTGCTGAACCAGTAGAAG-3′; IL-18 REV, 5′-GAGGCCGATTTCCTTGGTCA-3′; MYC FWD, 5′-CTTGAATGAAATGGAGAGC-3′; and MYC REV, 5′-GGAGCCTGCTTTTTTGTAC-3′. Prevalidated Prime-Time 6-carboxyfluorescein qPCR/probe sets obtained from IDT were used for the remaining genes. Comparison of mRNA expression levels between samples was performed using semiquantitative real-time RT-PCR (qPCR) with the Applied Biosystems sequence detection system according to the ΔΔ*C_T_* method (101), using GAPDH as a control.

### ELISA.

Supernatants harvested from treated cells were centrifuged at 400 × *g* for 4 min to remove cell debris and stored at −20°C prior to cytokine measurements. IL-1β was quantified using the BD OptEIA human IL-1β ELISA kit (BD Biosciences). IL-18 was quantified using the human IL-18 PicoKine ELISA kit (BosterBio). All measurements were made following the manufacturers’ protocols.

### Flow cytometry.

Cells to be analyzed via flow cytometry were collected using nonenzymatic Cellstripper (25-056-CI Corning Cellgro) and then pelleted at 1,800 × *g* at 4°C for 5 min. Samples were fixed with BD Cytofix/Cytoperm, incubated on ice for 15 min, pelleted, and then resuspended in 1× BD Perm/Wash containing the primary antibody. After a further 15-min incubation on ice, the samples were washed three times and then resuspended in 1× BD Perm/Wash containing secondary antibody. After a subsequent 15 min on ice, they were washed in 1× BD Perm/Wash and finally analyzed on a BD LSR II flow cytometer (BD Biosciences). Data obtained from the flow cytometer were analyzed using FlowJo version 9 (Tree Star).

### Indirect immunofluorescence assay.

Differentiated THP-1 cells were grown on coverslips in 24-well plates and treated as described in the text. At room temperature, cells were washed twice with PBS, fixed for 30 min in 3.7% formaldehyde, washed, and quenched for 10 min using 50 mM NH_4_Cl. Cells were permeabilized with 0.1% Triton X-100 for 7 min and washed three times with PBS containing 2% BSA. Cells were incubated with primary IE-reactive antibody in PBS containing 2% BSA at 37°C for 1 h, washed three times in PBS containing 2% BSA (10 min for each wash), and incubated with fluorescently labeled secondary antibody diluted 1:1,000 in PBS containing 2% BSA for 1 h. Cells were washed twice in PBS containing 2% BSA (10 min for each wash) and once in PBS. The secondary antibody used was goat anti-mouse 488 antibody (Invitrogen). Coverslips were mounted on a microscope slide with Vectashield mounting medium (Vector Laboratories, Burlingame, CA) containing 4,6-diamidino-2-phenylindole (DAPI).
